# Application of a Pickering Emulsion Stabilized by Zein and Cellulose Nanocrystalline Composite Particles to Preserve Kiwifruit

**DOI:** 10.3390/molecules30173478

**Published:** 2025-08-24

**Authors:** Yiping Liu, Weixiang Qiu, Yalan Mo, Jing Tian, Muxiang Liao, Binghong Jia, Qian Zhou, Feichi Liu, Xiaogang Li

**Affiliations:** 1 Institute of Cotton and Sericulture, Hunan Academy of Agricultural Sciences, Changsha 410127, China; ypliu619@hotmail.com (Y.L.); liaomuxiang@hotmail.com (M.L.); binghongjia@hotmail.com (B.J.); zhouqian901010@hotmail.com (Q.Z.); 2 Engineering & Technology Research Center for Bio Pesticide and Formulating Processing, College of Plant Protection, Hunan Agricultural University, Changsha 410128, China; qiuwx@stu.hunau.edu.cn (W.Q.); myl12@stu.hunau.edu.cn (Y.M.); tianjing199802@hotmail.com (J.T.)

**Keywords:** zein colloidal particle, cellulose nanocrystals, Pickering emulsion, carvacrol, kiwifruit preservation, safety assessment

## Abstract

This study involved developing a Pickering emulsion system based on a composite material comprising zein colloidal particles (ZCPs) and cellulose nanocrystals (CNCs) with the aim of exploring its potential application in fruit preservation by loading carvacrol (CAR). The system (CAR@ZCPE) consists of ZCP particles with an average size of approximately 317 nm in a composite with CNC particles of approximately 85 nm at an optimal mass ratio (ZCP/CNC = 1:3) to form stable particles encapsulating CAR. The results indicate that CAR@ZCPE is an O/W Pickering emulsion that can be diluted indefinitely in water and exhibits excellent environmental stability. Rheological analysis revealed that it exhibits shear-thinning properties and a gel-like network structure, which explains its good stability. Bioactivity evaluation revealed that CAR@ZCPE exhibited inhibitory activity against *Botryosphaeria dothidea*, with an inhibition rate of 63.60% at a concentration of 50 mg/L. Kiwifruit preservation experiments confirmed that CAR@ZCPE significantly reduced the degree of kiwifruit decay, and cell activity evaluations confirmed its biosafety. The total apoptotic rate of LO2 cells was 2.10%, indicating that the emulsion did not affect the cell growth cycle. This study successfully developed a CAR Pickering emulsion stabilized by ZCP-CNC composite particles. This emulsion system combines high stability, excellent antibacterial activity, and excellent biocompatibility. Kiwifruit preservation experiments validated its potential as a safe and efficient new preservative, providing an innovative method for preserving fruits using ZCP-CNC-composite-stabilized Pickering emulsions.

## 1. Introduction

Kiwifruit (*Actinidia chinensis* Planch.) is a soft, tender, and juicy fruit that is popular owing to its unique flavor, high nutrient content, health benefits, and medicinal properties [1,2]. However, kiwifruits are susceptible to decay during post-harvest storage and transportation, which significantly reduces their quality and storage time. Soft rot, which is caused by the fungus *Botryosphaeria dothidea*, is the primary cause of kiwifruit rot, often resulting in complete rot and economic losses [3]. Currently, soft rot control relies on physical and chemical treatments, including the regulation of storage temperature and the use of traditional fungicides [4,5]. However, traditional fungicides have several limitations. For example, commonly used chemicals are hazardous, and biologics are unstable and susceptible to environmental factors. This instability can result in significant losses during application, with a limited amount of active ingredients reaching the intended target, resulting in reduced pesticide efficacy, low utilization rates, and the increased risk of resistance development [6,7,8].

Pickering emulsions have attracted the interest of researchers investigating fruit preservation owing to their safety and stability. These systems are stabilized by solid particles rather than surfactants because the solid particles anchor to the oil–water interface, forming a dense three-dimensional barrier that provides long-term stability by resisting agglomeration and Ostwald maturation [9,10,11]. Furthermore, particle-stabilized emulsions exhibit greater resistance to the evaporation of volatile oils than surfactant-stabilized emulsions [12]. Wei et al. stabilized cinnamaldehyde Pickering emulsions using cellulose nanofibers as a novel essential oil delivery system, demonstrating their broad applicability in mango preservation strategies [13]. Moreover, Pu et al. prepared composite essential oil Pickering emulsions stabilized with zein–gum arabic nanoparticles, achieving excellent preservation results when storing pork and “Yellow Crown” pears [14]. Thus, Pickering emulsions have promising applications for inhibiting the propagation of pathogenic microorganisms in food products, resulting in bacteriostatic and preservative effects.

Zein was first discovered by Gorham in 1821 as the primary storage protein found in corn. It is a non-toxic natural macromolecule, accounting for 30% to 60% of the total protein content. The high degree of hydrophobicity exhibited by zein is attributable to the prevalence of a substantial number of hydrophobic α-helical structures and hydrophobic amino acids. However, this protein also possesses the capacity to dissolve in ethanol solutions of suitable concentrations [15,16,17]. The preparation methods for zein colloidal particles (ZCPs) are diverse, commonly employing techniques such as antisolvent precipitation, solvent evaporation, pH-driven processes, and chemical cross-linking to facilitate the self-assembly of zein into zein colloidal particles. Owing to their excellent bio-compatibility and bio-adhesion properties, ZCPs are ideal stabilizers for use in food delivery systems and biomedical therapeutics [18,19]. De Folter et al. reported the use of ZCPs as natural interfacial stabilizers in the preparation of O/W PE [20]. Cellulose is considered to be one of the most widely used biopolymers. It is a natural, sustainable, low-cost, and highly safe material, which makes it a highly competitive natural raw material. Cellulose nanocrystals (CNCs) are highly crystalline rod-shaped nanoparticles with lengths ranging from 50 to 300 nm and diameters of approximately 5 to 100 nm, which can be produced in stable aqueous suspensions [21]. The high aspect ratio of CNCs results in superior mechanical properties, thermal stability, and unique rheological and optical characteristics and facilitates interconnectivity during film formation and interface adsorption processes. The extensive applications of cellulose nanocrystals in fields such as biomedicine, packaging materials, and personal care products are attributable to the following advantages and environmental sustainability [22,23]. In particular, the ZCP-CNC composite system is widely used in Pickering emulsion solid-particle delivery systems for bioactive substances owing to its natural origin and good biocompatibility. For example, Wei et al. stabilized a Pickering emulsion by combining ZCPs and CNCs, while also maintaining its bio-accessibility and stability, to deliver β-carotene [24]. At present, Pickering emulsions stabilized by ZCP and CNC composite particles are chiefly concerned with interfacial stability and the delivery of food components and have not yet been applied in the preservation of fruit. Consequently, the utilization of this system for the post-harvest preservation of kiwifruit holds promising application prospects. Carvacrol (CAR) is a natural monoterpene phenol that exhibits antimicrobial, antioxidant, anticarcinogenic, and antiseptic properties [25,26]. Preliminary laboratory screening has revealed that CAR exhibits high bacteriostatic activity against polymyxins, suggesting its potential for controlling soft rot disease in kiwifruit.

In this study, Pickering emulsions with composite interfaces were prepared by coating CAR onto CNCs and ZCPs. The optimal mass ratio of the blends was determined using several parameters, and the properties of the emulsions were investigated using a rheometer and through storage at different temperatures, pH values, ionic strength values, and dilutions. The antibacterial activities of the emulsions were evaluated using the mycelial growth rate method, and their effect on kiwifruit freshness was investigated. The safety of the emulsions was also evaluated using cell activity and apoptosis tests. The present study constructed a Pickering emulsion of ZCP-CNCs stabilized with carvacrol, thereby expanding the potential application of ZCP-CNC composite particles in the field of fruit preservation and providing a new and effective strategy for the prevention and control of post-harvest diseases in kiwifruit.

## 2. Results and Discussion

### 2.1. Characterization of ZCP and CNC

**Figure 1:**
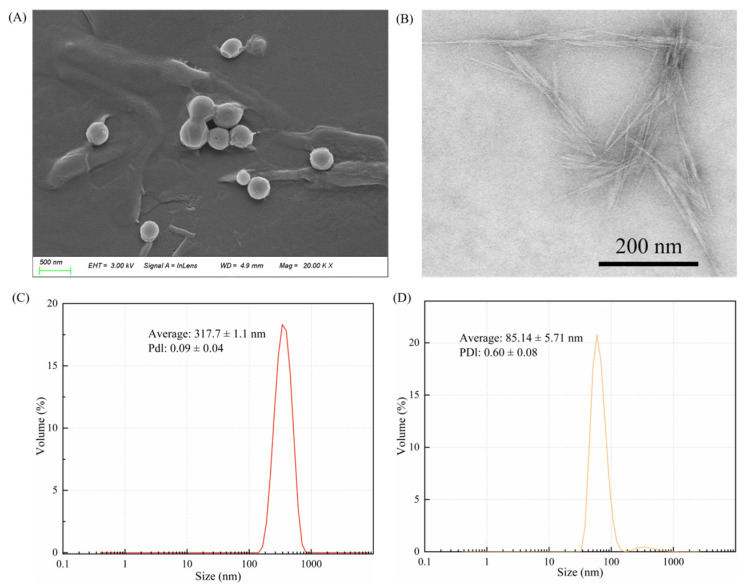
(**A**) Micromorphology of the ZCPs observed using SEM and (**B**) CNCs detected using TEM. DLS for (**C**) ZCPs and (**D**) CNCs.

The ZCPs were spherical in shape and approximately 317 nm in size. They exhibited high polydispersity in terms of particle size (Figure 1A,C). Owing to their inherent hydrophobicity, slight aggregation between the particles was observed. The large size of the ZCPs limited their adsorption rate at the oil–water interface. However, the resulting emulsion was highly stable owing to its high desorption energy [24]. The size of the ZCPs was affected by the ratio of ethanol-to-water, mixing time, speed, and rate at which corn-alcohol-soluble protein particles were added [27]. Figure 1B shows a transmission electron microscopy (TEM) image of the CNC particles, which appear hard and needle-like, and the average particle size, as measured by DLS, is around 85 nm (Figure 1D). The particles exhibited compact reticulation because of their high aspect ratio.

### 2.2. Screening of Different Pickering Emulsions (CAR@ZCPEs)

**Figure 2:**
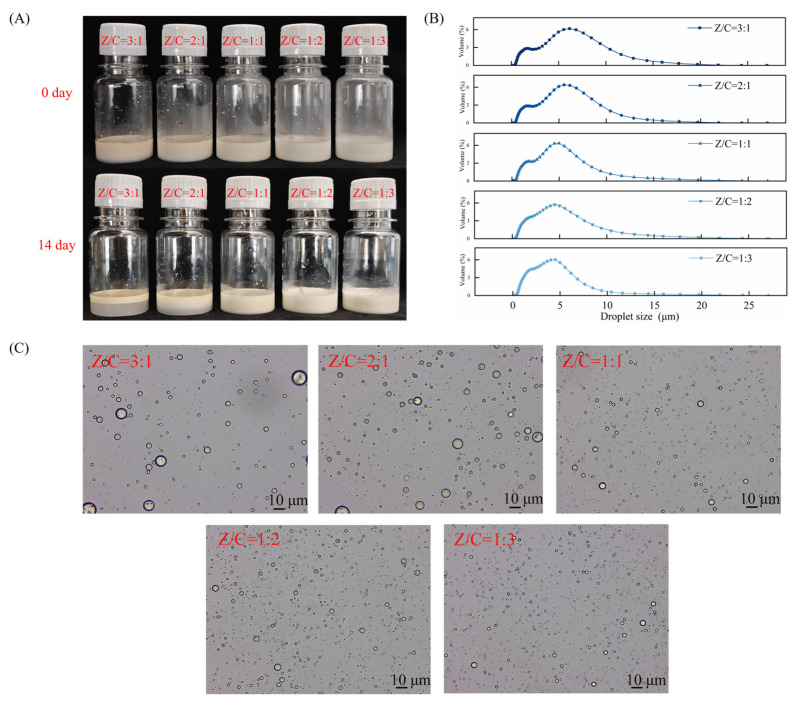
(**A**) Images of the Pickering emulsion prepared with different ZCP/CNC mass ratios on days 0 and 14; (**B**) droplet size distribution; and (**C**) optical microscopic images.

Emulsification and emulsion stability indicate the ability of composite particles to form and stabilize emulsions. In this study, Pickering emulsions were prepared by blending different ZCP/CNC mass ratios at room temperature, and their stable states were observed on days 0 and 14. As shown in Figure 2A, the emulsions with ZCP/CNC ratios of 3:1 and 2:1 exhibited water precipitation with storage time, resulting in delamination. Their creaming indices were calculated to be 77.52 and 67.42, respectively (The data are shown in Table 1). Emulsions with ZCP/CNC ratios of 1:1, 1:2, and 1:3 were not delaminated. This is due to the increased CNC content of the composite nanoparticles. Their rod-like structure enables them to form a denser membrane at the interface, thereby stabilizing the emulsion [28]. As shown in Figure 2B, the droplet size peak of the emulsion gradually decreased as the mixing ratio of CNCs increased, and the droplet size distribution tended to contain only a single peak. This was due to the larger size of the protein particles, which formed larger aggregates through electrostatic adsorption with CNCs, resulting in additional peaks in the particle size measurements. The optical microscope images (Figure 2C) revealed that a high percentage of CNCs resulted in the formation of fine droplets. Based on stability, droplet size distribution, and the state of droplets under microscope., a Pickering emulsion with a ZCP/CNC ratio of 1:3 was selected for subsequent studies and labeled as CAR@ZCPE.

### 2.3. Dilution State Analysis

**Figure 3:**
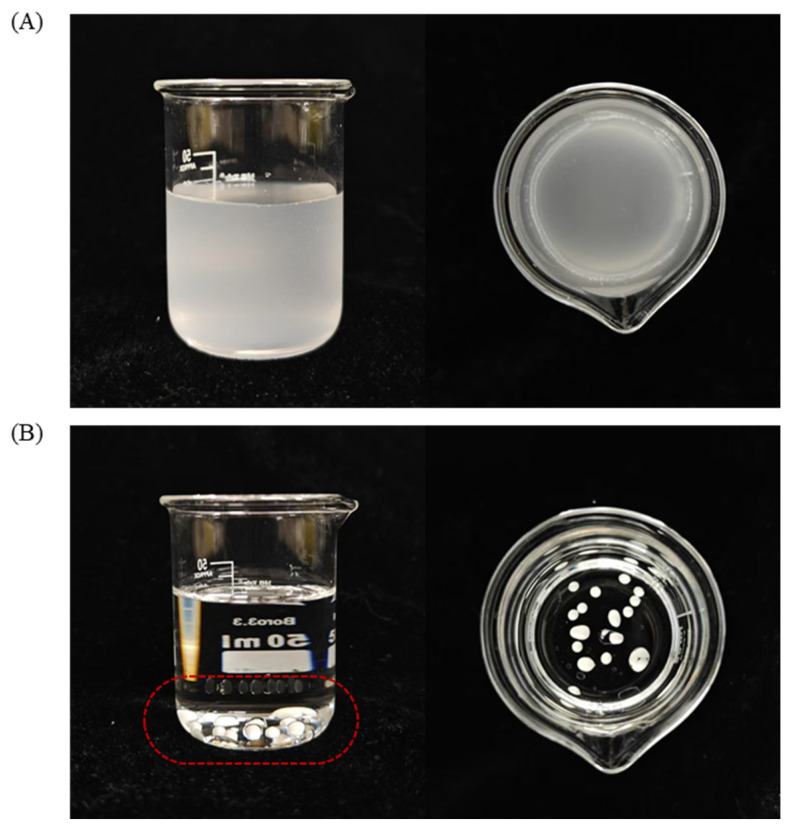
Dispersion states of CAR@ZCPE in (**A**) water and (**B**) turpentine (The droplet is displayed within the red box).

In applications such as coating, impregnation, and preservation, Pickering emulsions are often diluted to achieve the desired concentration. Therefore, the dispersibility of emulsions plays a crucial role in this process. According to Figure 3, the CAR@ZCPE was diluted indefinitely in water to form a homogeneous and stable solution. However, the CAR@ZCPE remained in the form of liquid droplets in turpentine and did not disperse uniformly, indicating that it existed as an oil-in-water (O/W) emulsion. During dilution, the CAR@ZCPE droplets diffused into water to form a uniform suspension. This excellent dispersion was attributed to the unique structure of CAR@ZCPE and the tight anchoring of the fiber–protein composite particles on the surface of the oil droplets [12]. The diluted emulsions were stabilized for at least 2 h, which is sufficient for most applications.

### 2.4. Emulsion Stability of CAR@ZCPE

**Figure 4:**
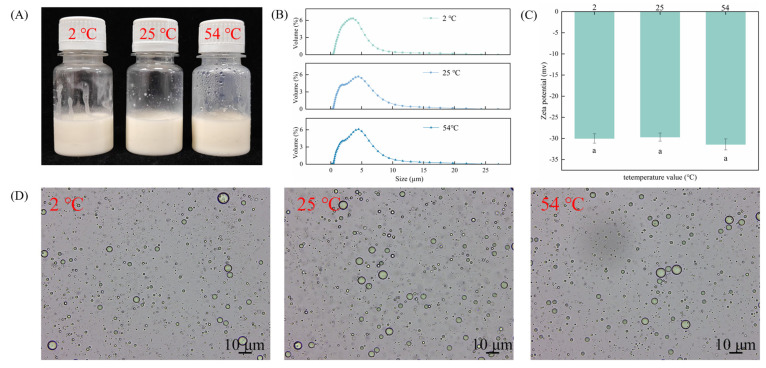
(**A**) Appearance of CAR@ZCPE following storage at different temperatures; (**B**) mean droplet diameter; (**C**) zeta potential; and (**D**) optical microscopic images. Different letters indicate significant differences according to the LSD test (*p*-value < 0.05).

The heating process increases the frequency of particle collisions, resulting in the exposure of hydrophobic regions of the protein and subsequent aggregation. To investigate the stability of CAR@ZCPE, it was stored at three different temperatures. The experimental results show that the Pickering emulsion loaded with CAR exhibits excellent chemical stability across the tested temperature range thanks to the synergistic stabilizing effects of CNCs and ZCPs. Even when stored at 54 °C, no significant emulsion separation or carvacrol degradation was observed, and the carvacrol retention rate in the emulsion remained high. These results demonstrate the effectiveness of this stabilization system under high-temperature conditions (Figure 4A). The average droplet size (Figure 4B) increased with temperature but remained stable. The zeta potential results indicate that the structure of solid particles was not destroyed by high temperatures, enabling their charges to remain stable (Figure 4C). This further demonstrates the excellent chemical stability of emulsions at high temperatures. Optical microscopy images (Figure 4D) revealed that the emulsion droplets remained spherical without deformation or precipitation of particles. This was due to the strong adsorption of the ZCP–CNC particles at the oil–water interface, creating a spatial barrier that prevented droplet aggregation [29]. Thus, the CAR@ZCPE exhibited strong anti-agglomeration properties during heat treatment and remained stable over a wide temperature range.

**Figure 5:**
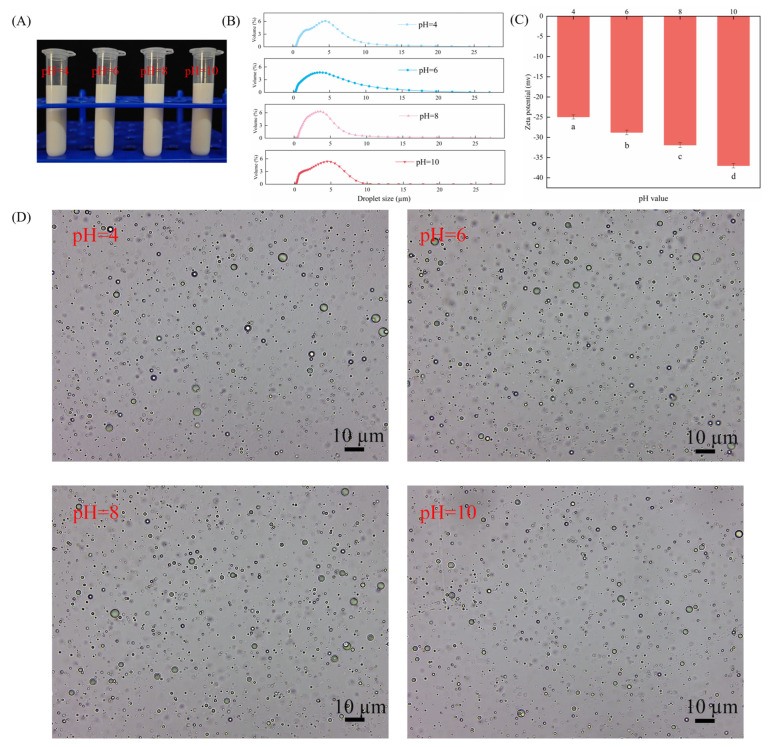
(**A**) Appearance of CAR@ZCPE following storage at different pH values; (**B**) mean droplet diameter; (**C**) zeta potential; and (**D**) optical microscopic images. Different letters indicate significant differences according to the LSD test (*p*-value < 0.05).

It has been demonstrated that the pH of the medium exerts an influence on the charge of particles, thereby affecting emulsion stability. Figure 5 investigates the effect of varying pH levels on the stability of Pickering emulsions with particle-to-particle interface mixing. As demonstrated in Figure 5A, emulsions have been shown to remain stable at varying pH values, with no indication of phase separation. It is evident from Figure 5B,D that an increase in pH results in a decrease in droplet size while ensuring effective dispersion of the droplets. This phenomenon can be attributed to the increased zeta potential that results in the strengthening of electrostatic repulsion between droplets (Figure 5C). Consequently, this provides sufficient space and electrostatic repulsion to maintain the stability of Pickering emulsions with particle–particle interface complexes.

**Figure 6:**
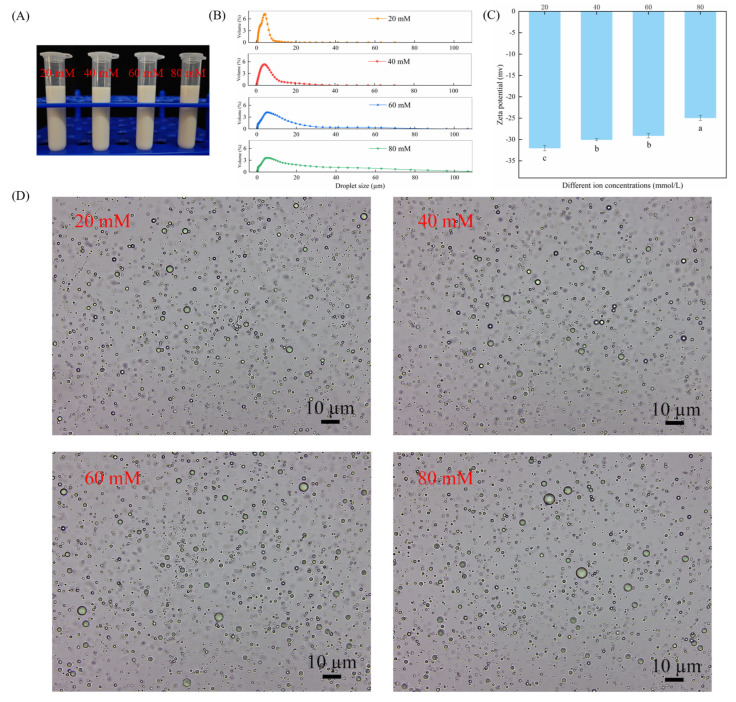
(**A**) Appearance of CAR@ZCPE following storage at different ion concentrations; (**B**) mean droplet diameter; (**C**) zeta potential; and (**D**) optical microscopic images. Different letters indicate significant differences according to the LSD test (*p*-value < 0.05).

In order to ascertain the effect of ionic strength on the stability of the CAR@ZCPE, different amounts of NaCl were added. The appearance of the CAR@ZCPE is demonstrated in Figure 6A. Phase separation or decomposition was not observed in the emulsion. As NaCl was added, the droplet size increased (Figure 6B), and the absolute value of the zeta potential decreased (Figure 6C). As demonstrated in Figure 6D, it is evident that the droplets underwent an increase in size, and under conditions of high ionic strength, a propensity for aggregation was observed. This phenomenon can be attributed to the weakening of electrostatic repulsion between droplets, a consequence of electrostatic shielding, and the occurrence of hydrophobic attraction between particles, resulting in droplet flocculation [30].

### 2.5. Rheological Properties of CAR@ZCPE

**Figure 7:**
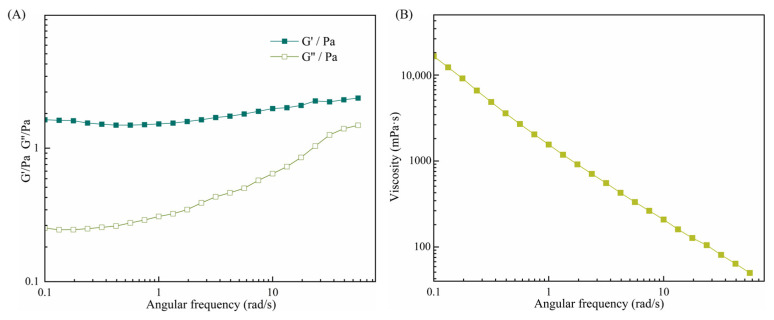
(**A**) Effects of shear frequency on the elastic storage modulus (G′) and loss modulus (G″) of the CAR@ZCPE (**B**) and on the apparent viscosity of the CAR@ZCPE.

In order to further investigate the structural properties of the CAR@ZCPE, this study characterized the rheological behavior of the ZCP–CNC-stabilized CAR@ZCPE by performing frequency and shear rate scans (Figure 7A). In the angular frequency scanning experiments, the CAR@ZCPE was evaluated by applying a fixed stress within the linear response region. The energy storage modulus (G′) of the CAR@ZCPE exhibited a consistent predominance over the loss modulus (G’’), thereby suggesting that the CAR@ZCPE displayed an elastic gel-like structure [31]. This structural property was attributed to the particle barrier layer formed by ZCP-CNCs at the oil–water interface, as well as the three-dimensional network structure formed within the continuous phase.

The shear rate scanning experiments revealed that the apparent viscosity of CAR@ZCPE significantly decreased with increasing shear rate, indicating that the emulsion was a non-Newtonian pseudoplastic fluid with typical shear-thinning behavior [32]. This rheological property can be explained by the deformation of the mesh structure formed in the equilibrium state under shear stress. For example, an increase in shear stress caused the three-dimensional mesh structure to rupture and intensified the deformation of the droplets, thereby decreasing the apparent viscosity of the CAR@ZCPE [33]. This rheological characterization is important for gaining a deeper understanding of the physical properties of CAR@ZCPE, including its appearance and stability.

### 2.6. Antibacterial Activity of CAR@ZCPE

**Figure 8:**
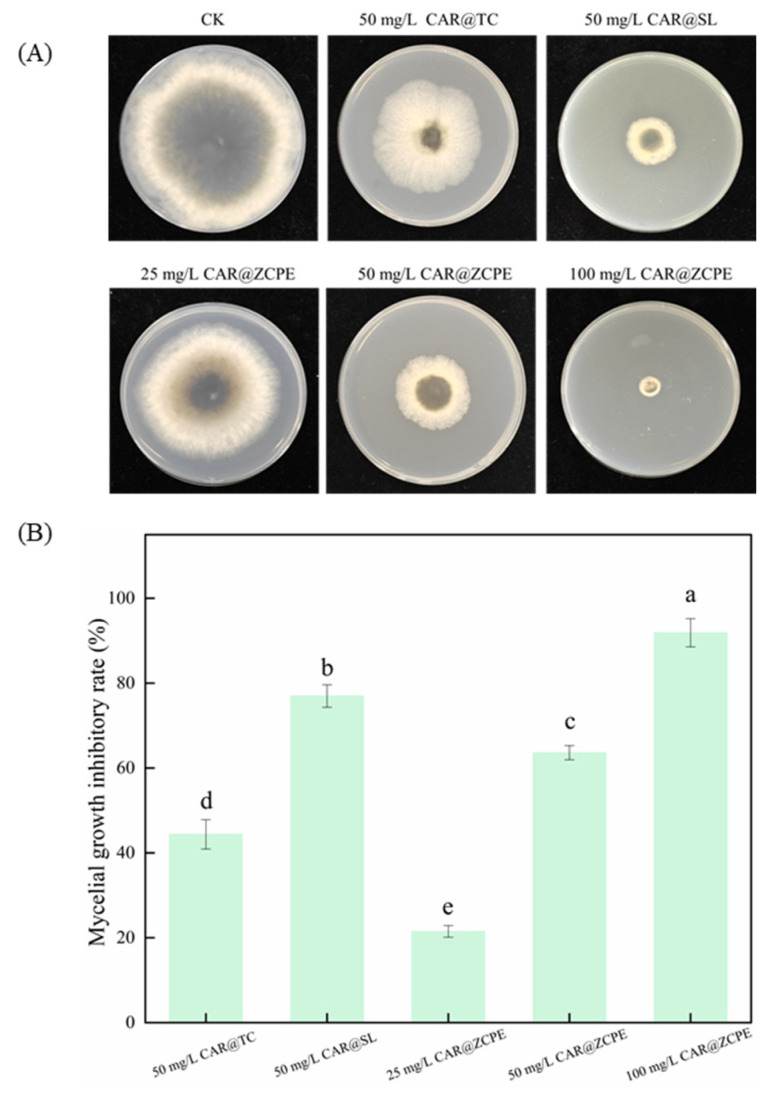
(**A**) Mycelial growth of *B. dothidea* on PDA plates containing CAR@ZCPE and (**B**) growth inhibition rate. Different letters indicate significant differences according to the LSD test (*p*-value < 0.05).

Pickering emulsions were prepared using CAR as a model pesticide and CNCs and ZCPs as stabilizers. The inhibitory activity of the CAR@ZCPE was evaluated by measuring the mycelial growth rate of the emulsions against *B. dothidea* (Figure 8A). The mycelium growth inhibition rates were 21.49%, 63.60%, and 91.88% on potato dextrose agar (PDA) plates with CAR@ZCPEs at final concentrations of 25, 50, and 100 mg/L, respectively (Figure 8B). Consequently, the inhibition rate exhibited an upward trend in proportion to increasing concentrations. Compared to the control, the mycelial growth inhibition of CAR@ZCPE at a low concentration was lower than that of the original drug (CAR alcohol; CAR@TC: 44.38% inhibition) and the 5% CAR alcohol soluble liquid (CAR@SL: 76.97% inhibition). The mycelial growth inhibition rate of the CAR@ZCPE was higher than that of CAR@TC and lower than that of CAR@SL at the same concentration because CAR is an essential oil with high volatility. However, at the same concentration, the CAR in the CAR@ZCPE was encapsulated by CNCs and ZCPs, which impeded its volatilization, resulting in a higher inhibition rate of mycelial growth in the CAR@ZCPE than in CAR@TC. Furthermore, the mycelial growth inhibition rate of CAR@SL was higher than that of CAR@TC. As a commercially available soluble formulation, CAR@SL contains several chemical additives. Even without a substance coating to impede the volatilization of CAR, these chemicals had an inhibitory effect on *B. dothidea*, ultimately leading to the highest inhibition rate of mycelial growth in CAR@SL at the same concentration. Thus, the incorporation of the CAR@ZCPE had a significant inhibitory effect on the mycelial growth of *B. dothidea* at all concentrations.

### 2.7. Fruit Preservation

**Figure 9:**
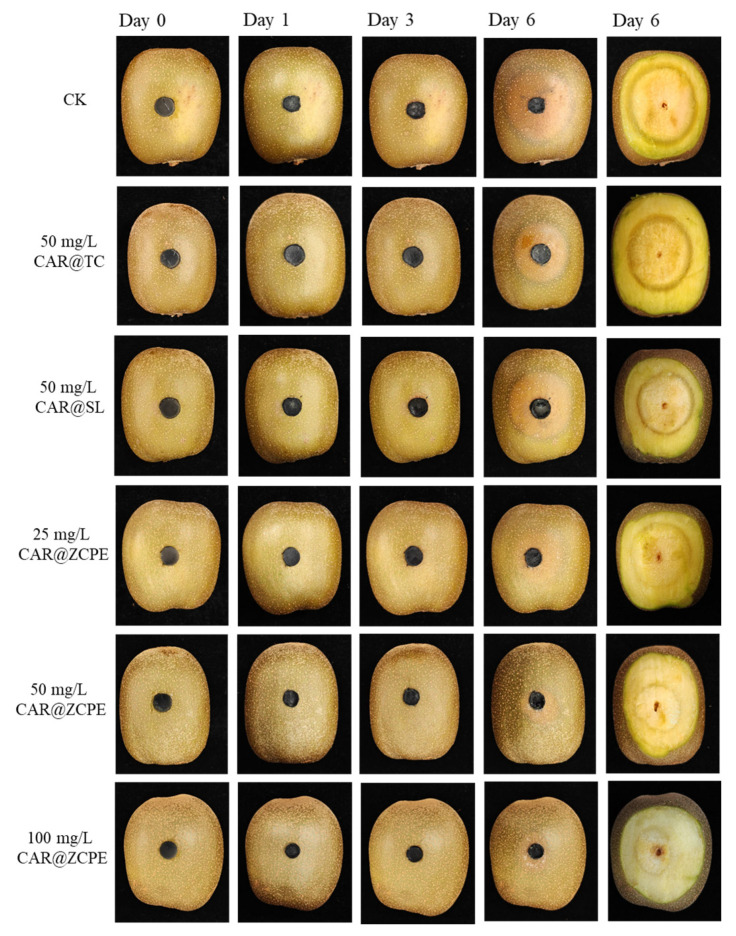
Effects of CAR@TC, CAR@SL, and CAR@ZCPE treatment on the post-harvest decay of kiwifruit inoculated with *B. dothidea* stored at 25 °C.

**Figure 10:**
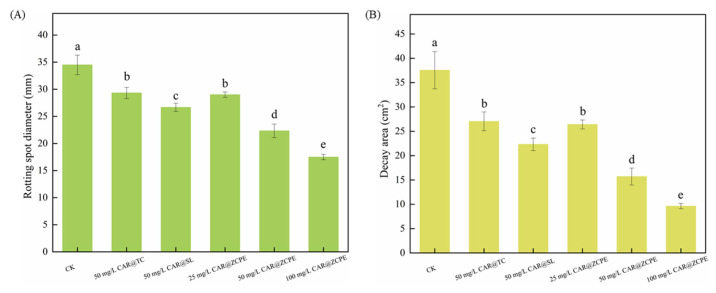
Diameters of the (**A**) rot spots and (**B**) areas of decay of kiwifruits treated with CAR@TC, CAR@SL, and CAR@ZCPE stored at 25 °C for 6 days. Different letters indicate significant differences according to the LSD test (*p*-value < 0.05).

The impact of the emulsions on kiwifruit freshness was evaluated through an ex vivo test. Figure 9 and Figure 10 show the extent of fruit rot and the diameters and areas of rot spots caused by a *B. dothidea* invasion in kiwifruit treated with CAR@TC, CAR@SL, and CAR@ZCPE during storage at 25 °C. During the initial three-day period, no visible lesions were observed on the surface of the fruit. However, after a six-day period, prominent water-soaked lesions became apparent on the fruit. These lesions were circular and radiated outwards from the fungal cakes. A light black halo was present at the periphery of the lesions in fruits treated with CAR@TC and CAR@SL, whereas CAR@ZCPE-treated fruits did not present halos. Thus, the CAR@ZCPE significantly inhibited *B. dothidea* infestation. A cross-sectional view of the fruit was obtained by cutting it through the outer surface to observe internal changes. The lesion areas of fruits treated with the CK, CAR@TC, and CAR@SL exhibited severe softening, accompanied by extensive decay and loss of pulp elasticity.

The pulp had a pasty or semiliquid texture and emitted a strong, sour, and rotting odor. The fruit decay spots of those treated with CK, CAR@TC, and CAR@SL had diameters of 34.50, 29.33, and 26.67 mm over decay areas of 37.55, 27.04, and 22.33 cm^2^, respectively. Fruits treated with the CAR@ZCPE showed decay at a concentration of 25 mg/L, with decay spots and areas measuring 29.00 mm and 26.41 cm^2^, respectively. These results were comparable to those of the CAR@TC treatment. Thus, encapsulating CAR with CNCs and ZCPs improved its stability, enabling the CAR@ZCPE at a concentration of 25 mg/L to produce results similar to those of CAR@TC at a concentration of 50 mg/L. Moreover, after treatment with 50 mg/L CAR@ZCPE, the degree of decay further decreased. The diameter of the decay spot was 22.33 mm, and the decay area was 15.70 cm^2^, which were significantly lower than those in all control groups. At the same concentration, CAR@SL exhibited better preservation than CAR@TC owing to the chemical additives present in CAR@SL. However, the limitations of the formulation type and the high volatility of CAR resulted in a lower concentration of the active ingredient in CAR@SL, which was ineffective in inhibiting the growth of *B. dothidea*. Because the CAR@ZCPE is a Pickering emulsion system, the adsorption of solid particles at the interface of the two phases effectively impeded the volatilization of essential oils. This resulted in superior freshness preservation of fruits treated with the CAR@ZCPE compared to other formulation types at the same concentration. At a concentration of 100 mg/L, the spot diameter was 17.50 mm, and the softened area was 9.62 cm^2^. The fruit showed only slight softening and no significant decay, which could be attributed to water loss owing to the lack of pericarp protection caused by physical damage to the fruit. Thus, CAR@ZCPE impeded the volatilization of CAR, effectively preventing the post-harvest decay of kiwifruit caused by *B. dothidea* infection and outperforming both the original and soluble CAR formulations at the same treatment concentration.

Salma et al. developed a film containing an halloysite–chitosan–polyvinyl alcohol gel with thymol with the purpose of preserving kiwifruit and extending its shelf life [34]. Li et al. developed an edible metal–organic framework with excellent ethylene gas adsorption properties, which delayed the ripening of red kiwifruit and extended its shelf life [35]. This study investigated the preservation effect of Pickering emulsions on kiwifruit, which were achieved via spraying a composite film or material absorption, as well as kiwifruit freshness. The results of this study provide a foundation for enhancing the freshness of fruits by combining various methods to increase their storage periods.

### 2.8. Biological Safety Evaluation

**Figure 11:**
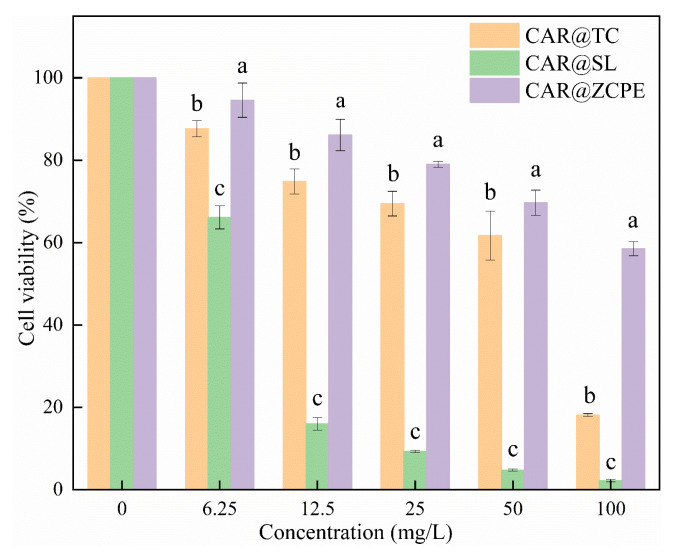
Cell viabilities of LO2 cells treated with different concentrations of CAR@TC, CAR@SL, and CAR@ZCPE. Different letters indicate significant differences according to the LSD test (*p*-value < 0.05).

During the application process, pesticides can easily enter the body of the applicator; therefore, their effects on human cells must be considered. The effect of the CAR@ZCPE on LO2 human hepatocytes was examined using a cell proliferation assay. As demonstrated in Figure 11, there was a decline in cell viability with increasing concentrations, both within the control group and the treated group. Across the concentration gradient, cell viability ranged from 18.12% to 87.68% following treatment with CAR@TC, while that for CAR@SL ranged from 2.21% to 66.14%. In contrast, the cell viability after treatment with the CAR@ZCPE ranged from 58.53% to 94.57%. Thus, cell viability was significantly higher in the CAR@ZCPE-treated cells than in control cells at all concentrations tested. Unlike the original drug, solid particles formed an interfacial membrane that affected the direct contact between CAR and the cells, enhancing the safety of the emulsion. In contrast, soluble formulations contain various chemicals that greatly affect cell viability, thereby rendering them unsuitable for food preservation.

**Figure 12:**
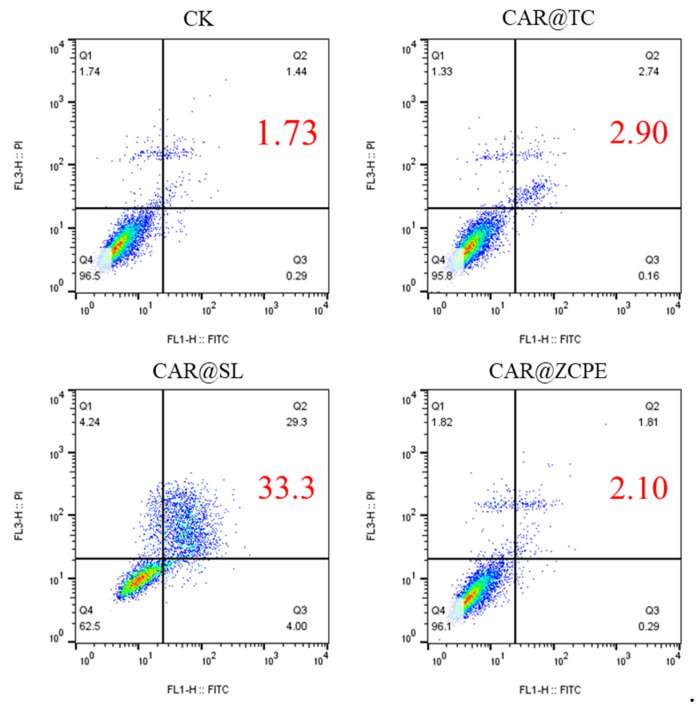
Cell apoptosis assays of LO2 cells treated with CAR@TC, CAR@SL, and CAR@ZCPE at a concentration of 25 mg/L.

To further evaluate the effect of the CAR@ZCPE on cell activity, the distribution status and apoptosis of drug-treated cells in each cycle were examined using flow cytometry. Figure 12 illustrates the results of apoptosis tests for CAR@TC-, CAR@SL-, and CAR@ZCPE-treated human LO2 hepatocytes. The overall apoptosis rate in the CAR@TC treatment group was 2.90%, with most cells in the Q4 living cell phase. In contrast, the overall apoptosis rate following CAR@SL treatment was 33.3%, with a significant proportion of cells in the Q2 late apoptosis phase. The CAR@ZCPE treatment group exhibited an overall apoptosis rate of 2.10%, which was lower than that of the control group, suggesting that CAR@ZCPE is safe for cells. Feng et al. prepared pyriproxyfen nanoemulsions and observed a significant decrease in the apoptosis of LO2 cells. Thus, reducing the droplet size of an emulsion can reduce its toxicity [36]. In contrast, the present study used particle adsorption to prepare a Pickering emulsion system that reduced the apoptotic effect of the formulation on human cells, thereby improving its safety.

## 3. Materials and Methods

### 3.1. Materials

Zein was procured from Macklin Biochemical Co., Ltd. (Shanghai, China), CNCs were supplied by ScienceK Co., Ltd. (Shanghai, China), and CAR was supplied by Huabang Natural Spice Co., Ltd. (Jiangxi, China). CAR@SL was procured from the Dewei Herbal Biotechnology Co., Ltd. (Shanxi, China). Sodium hypochlorite, anhydrous ethanol, sodium chloride, sodium hydroxide, and hydrochloric acid (36–38%) were supplied by Sinopharm Group Chemical Reagent Co., Ltd. (Shanghai, China).

### 3.2. Preparation of Suspensions of the ZCPs and CNCs

Suspensions of the ZCPs and CNCs were prepared according to a previously de-scribed method, with certain modifications [37]. First, 1.0 g of zein was dissolved in 30 mL of an 80% (*v*/*v*) aqueous ethanol solution and stirred at 25 °C at 500 rpm for 30 min. Slowly, 75 mL of deionized water was added, and the mixture was continuously stirred for 2 h. The ethanol in the solution was removed using rotary evaporation at 45 °C, followed by mixing to 100 mL using deionized water. The resulting ZCP suspension was adjusted to pH 4.0 using 0.1 M HCl. Another 1.0 g of CNC powder was dispersed in 100 mL of deionized water and sonicated for 20 min to obtain a 1% (*w*/*v*) CNC suspension, and the pH was adjusted to 4.0 using 0.1 M HCl or NaOH. In all samples, 50 mM NaCl was maintained in the aqueous phase to partially shield the CNC surface from charge and promote interfacial filling.

### 3.3. Characterization of the ZCP and CNC

The ZCP dispersion was diluted 10-20 times with water, and a drop of the dispersion was added to a silicon wafer. The wafer was dried at room temperature and bonded to conductive adhesive spray gold. Scanning electron microscopy (SEM; Sigma 300, Carl Zeiss, Oberkochen, Germany) was used to observe the morphology. The ZCP dispersion was diluted to 0.1% (*w*/*v*) in water then sonicated until fully dispersed. The protein particle size and polydispersity index were detected using dynamic light scattering (DLS, Zetasizer Nano ZS90, Worcestershire, United Kingdom).

The morphologies of the CNC were determined using TEM (HT7800, Ruli, Tokyo, Japan). The samples were diluted to 0.01 mg/mL with purified water, and a drop (20 μL) of the dispersion was added to the copper grid of the transmission electron microscope. The CNCs were stained with a uranyl acetate solution for 30 s. Excess uranyl acetate solution was then absorbed using filter paper, following by rinsing with water and drying at room temperature. The resulting sample was recorded for morphology. The CNC dispersion was diluted to 0.1% (*w*/*v*) in water then sonicated until fully dispersed. The particle size and polydispersity index were examined using DLS.

### 3.4. Preparation and Screening of the Pickering Emulsion (CAR@ZCPE)

#### 3.4.1. Preparation of CAR@ZCPE

**Figure 13:**
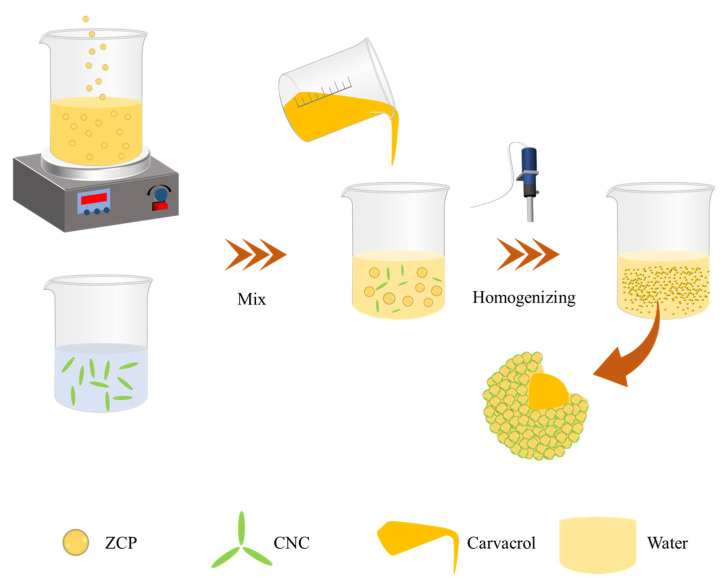
Schematic representation of the fabrication process of the CAR@ZCPE.

The ZCPs and CNCs were mixed at different mass ratios (3:1, 2:1, 1:1, 1:2, and 1:3) to obtain a mixed dispersion. CAR was added to the mixed dispersion and emulsified for 5 min at 15,000 rpm using a digital homogenizer (T25, IKA, Stauffen, Germany) to prepare a Pickering emulsion with an oil-to-water ratio of 2:8 (*w*/*w*), which was stored at 25 °C until subsequent investigations (Figure 13).

#### 3.4.2. Screening of CAR@ZCPE

The dispersions of the ZCP/CNCs, which vary in mass ratio, were stored at 25 °C. The appearance of the emulsion was photographed on days 0 and 14, and the creaming index (CI) was calculated as follows Equation (1). A small amount of the sample was added dropwise to an automatic circulation dispersion feeder (BT-801, Dandong Best Instrument Co., Ltd. Dandong City, Liaoning Province, China) until the laser droplet shading rate reached 10%, resulting in a sample droplet size distribution graph (BT-9300S, Dandong Best Instrument Co., Ltd. Dandong City, Liaoning Province, China). After diluting the sample 10–20 times, droplet distribution was observed and photographed using an optical microscope. Based on the emulsion stable state, CI value, droplet size distribution, and microscope diagrams, the optimal mass ratio was screened as the subsequent research objective.

(1) $$CI\;(\%)=\frac{H_{s}}{H_{t}}\times 100$$

where Hs denotes the height of the serum and Ht denotes the total height of the emulsion.

### 3.5. CAR@ZCPE Dilution State Tests in Different Phases

The CAR@ZCPE (200 μL) was dispensed into deionized water and turpentine and rested at 25 °C for 2 h with gentle stirring. The dispersion state and stability of the diluted emulsion were observed and photographed.

### 3.6. Stability Test of CAR@ZCPE

To examine the stability of the CAR@ZCPE, the prepared emulsion was stored under sealed conditions at 54 ± 2, 25 ± 2, and 2 ± 2 °C for 14 days and the stable states, microscope diagrams, zeta potential, and mean droplet size distribution were obtained. To determine the effect of pH on the stability of the CAR@ZCPE, the emulsion pH (ranging from 4 to 10) was adjusted using configured 1 M HCl and 1 M NaOH solutions. After resting for 24 h, the stable states of the samples were observed, microscopic diagrams were obtained, and the mean droplet size distribution and zeta potential of the emulsions was determined. To determine the effect of ionic strength on the stability of the CAR@ZCPE. The final concentrations of 20, 40, 60, and 80 mM were achieved by adding NaCl. After resting for 24 h, the samples’ stable states and microscope diagrams of the samples were observed, and the mean droplet size distribution and zeta potential of the emulsions were determined.

### 3.7. Investigating the Rheological Properties of CAR@ZCPE

The rheological properties were determined using a rotational rheometer (Haak Mars60, Thermo, Waltham, Massachusetts, Germany) equipped with parallel plates (60 mm). Dynamic frequency sweep tests were performed in the range of 0.1–100 rad/s to obtain the apparent viscosity (η), energy storage modulus (G′), and loss modulus (G′′) values at 1.0% strain within the linear viscoelastic range.

### 3.8. Application of CAR@ZCPE in Kiwifruit Preservation

#### 3.8.1. In Vitro Bacterial Inhibition Assay

The in vitro inhibitory activity of the CAR@ZCPE against *B. dothidea* was determined using a mycelial growth rate assay with CAR@TC and CAR@SL as positive controls, according to a previous method, with certain modifications [38]. In particular, 8 mm diameter *B. dothidea* patties were placed on the surface of PDA plates containing different concentrations (25, 50, and 100 mg/L) of the CAR@ZCPE, with both CAR@TC and CAR@SL at a concentration of 50 mg/L. The inoculated PDA plates were incubated at 26 ± 2 °C for 5 days. Following incubation, the cross-method was used to determine the mean diameter of the radial mycelial growth using deionized water as a blank control, and cell viability was evaluated using Equation (2):

(2) $$Inhibition\;Rate\;(\%)=\frac{D_{control}-D_{treatment}}{D_{control}}\times 100$$

where D_control_ and D_treatment_ represent the mycelial growth diameter on the control and treated plates, respectively.

#### 3.8.2. Fruit Preservation Evaluation Experiment

Kiwifruit freshness was evaluated according to the method described by Gomez-Maldonado et al., with minor modifications [39]. Fresh kiwifruit “Hongyang” was rinsed with tap water, and after spraying with 75% alcohol, the fruits were sterilized via immersion in a 1.0% sodium hypochlorite (NaClO) solution for 1 min followed by exposure to a UV lamp for 30 min. Subsequently, a 5 mm deep wound was created on the equatorial surface of each fruit using a sterile needle, and 20 μL of the CAR@ZCPE at concentrations of 25, 50, and 100 mg/L were added to the wound. The fungal cake was covered over the wound, and the treated kiwifruits were placed in sterile polyethylene boxes stored at 25 °C for 6 days. Fruit decay was recorded on days 0, 1, 3, and 6, and spot diameters and decay areas were measured on day 6. The fruits inoculated with sterile water and 50 mg/L of CAR@TC and CAR@SL were used as controls.

### 3.9. Cell Activity Tests

To evaluate the effects of different sample concentrations on cell viability, cells were cultured normally in culture flasks, and the medium was aspirated, washed with PBS, and digested in trypsin. Subsequently, a complete medium containing 10% fetal bovine serum was added to ensure digestion completion, and the cells were aspirated. The cells were collected using centrifugation, counted using a cell counter plate, and diluted with the medium. Cells were inoculated at a density of 1 × 10^4^ cells/well (per 100 μL) into 96-well plates lined with the material and incubated in a 5% CO_2_ incubator at 37 °C for 24 h. Subsequently, different concentrations (6.25, 12.5, 25, 50, and 100 mg/L) of CAR@TC, CAR@SL, and CAR@ZCPE were added, followed by incubation for another 12 h. The cells were then incubated in a medium containing 10% fetal bovine serum and the ZCPE and incubated for another 12 h. The original medium was then aspirated, and 200 μL fresh Dulbecco′s Modified Eagle Medium containing 10% fetal bovine serum and 20 μL of a CCK-8 solution were added to each well. The wells were incubated for 2 h, and the absorbance at 450 nm was measured using an enzyme marker (EPOCH2, Berten Instruments, Waltham, MA, USA). Cell viability was evaluated using Equation (3):

(3) $$Cell\;viability\;(\%)=\frac{D_{treatment}-D_{blank}}{D_{control}-D_{blank}}\times 100$$

In this assay, the group containing the medium and cells served as the treatment group, whereas the group containing only the medium and CCK-8 served as the control group.

### 3.10. Apoptosis Tests

LO2 cells were treated with samples (25 mg/L) for 24 h, and the cells were then digested with trypsin. Blank groups were not subjected to sample treatment. After removing the supernatant using centrifugation, the fine-time post-cells were collected and resuspended in a centrifuge tube with PBS, followed by centrifugation at 2,000 rpm for 5 min. Subsequently, the cells were resuspended in 100 mL of PBS buffer, and 5 μL annexin V-FITC and 10 μL propidium iodide were added. The suspension was incubated under light protection for 15 min. The cells in the samples were detected and measured using flow cytometry (BD Calibur, Franklin Lakes, NJ, USA), and the data were analyzed using FlowJo-V10 software.

### 3.11. Statistical Analysis

All measurements were performed in triplicate, and the resulting data were ex-pressed as mean ± standard deviation. Statistical analyses were performed using SPSS Statistics 26.0 with one-way analysis of variance. Significance levels were evaluated using Duncan′s new complex polarity method (*p* < 0.05).

## 4. Conclusions

In this study, a Pickering emulsion (CAR@ZCPE) based on ZCP/CNC (mass ratio 1:3) composite particle stabilization was successfully developed for loading carvacrol (CAR). CAR@ZCPE is an O/W Pickering emulsion with infinite dilatability in water. It has excellent stability with respect to temperature, pH, and ionic strength and unique rheological properties in the gel state (energy storage modulus greater than loss modulus and shear thinning). The CAR@ZCPE exhibited significant antibacterial activity against *B. dothidea* (63.60% inhibition at the same concentration as the CAR@TC) and remained biologically active at low concentrations. In the context of kiwifruit preservation, the application of the CAR@ZCPE demonstrated to be effective in the inhibition of soft rot caused by *B. dothidea*, thereby extending the fruit′s freshness period. The cellular assay demonstrated that the CAR@ZCPE has good biosafety, as indicated by the high cell activity and the apoptosis rate of only 2.10%. The present study reveals that CAR loaded in a ZCP-CNC-stabilized Pickering emulsion can provide a new and effective strategy for the prevention and control of post-harvest diseases in kiwifruit. Furthermore, it expands the potential application of ZCP-CNC composite particles in the field of fruit preservation, which is a new approach for the development of safe and efficient fruit and vegetable preservation technology.

## Figures and Tables

**Table 1:** Creaming index (CI) for different mixing ratios under specified storage periods.

Z/C	Day 0	Day 14
3:1	0	77.52 ± 2.31 a
2:1	0	67.42 ± 1.52 b
1:1	0	0 c
1:2	0	0 c
1:3	0	0 c

Data (mean ± SD) with different letters in the same column indicate significant differences (*p*-value < 0.05).

## Data Availability

The datasets used and/or analyzed during the current study are available from the corresponding author on reasonable request.
